# Combination strategy exploration for prior treated recurrent or metastatic nasopharyngeal carcinoma in the era of immunotherapy

**DOI:** 10.1038/s41598-024-52326-7

**Published:** 2024-01-20

**Authors:** Yaofei Jiang, Chun Chen, Guoying Liu, Ting Fang, Nian Lu, Weixin Bei, Shuhui Dong, Wangzhong Li, Weixiong Xia, Hu Liang, Yanqun Xiang

**Affiliations:** 1 Department of Nasopharyngeal Carcinoma, Sun Yat-Sen University Cancer Center, State Key Laboratory of Oncology in South China, Guangdong Key Laboratory of Nasopharyngeal Carcinoma Diagnosis and Therapy, Guangdong Provincial Clinical Research Center for Cancer, Sun Yat-sen University Cancer Center, Guangzhou, 510060 People’s Republic of China; 2 Department of Nuclear Medicine, The Seventh Affiliated Hospital of Sun Yat-sen University, 628 Zhenyuan Road, Shenzhen, 518107 People’s Republic of China; 3 Guangdong Provincial Key Laboratory of Malignant Tumor Epigenetics and Gene Regulation, Medical Research Center, Department of Radiotherapy, Sun Yat-Sen Memorial Hospital, Sun Yat-Sen University, Guangzhou, People’s Republic of China; 4 Department of Radiology, Sun Yat-Sen University Cancer Center, State Key Laboratory of Oncology in South China, Guangdong Key Laboratory of Nasopharyngeal Carcinoma Diagnosis and Therapy, Guangdong Provincial Clinical Research Center for Cancer, Sun Yat-Sen University Cancer Center, Guangzhou, 510060 People’s Republic of China

**Keywords:** Cancer, Immunology, Oncology

## Abstract

To assess the efficacy and safety of the combination of immune checkpoint inhibitors (ICIs) and target therapy (anti-angiogenesis or EGFR inhibitors) as a second-line or subsequent treatment for recurrent or metastatic nasopharyngeal carcinoma (R/M NPC), we conducted a retrospective study. In this study, previously treated R/M NPC patients were administered one of the following treatment: ICIs combined with target therapy and chemotherapy (ITC), ICIs combined with target therapy alone (IT), ICIs combined with chemotherapy (IC), or chemotherapy alone (C). The primary endpoint under consideration was progression-free survival (PFS), while secondary endpoints included overall survival (OS), objective response rate (ORR), disease control rate (DCR), and safety measures. A total of 226 patients participated in this study, with 70 receiving the ITC regimen, 48 receiving IT, 48 treated with IC, and 60 undergoing C alone. The median PFS for the four cohorts was 20.67, 13.63, 12.47, and 7.93 months respectively. Notably, ITC regimen yielded the most favorable PFS among these cohorts. The ITC cohort exhibited a comparable tumor response and safety profile to the IT and IC cohorts (*p* > 0.05), but superior tumor response compared to the C cohort (*p* < 0.05). The ITC regimen also conferred a significant improvement in OS when comparing to C alone (HR 0.336, 95%CI 0.123–0.915, *p* = 0.033). The IT and IC regimens achieved a nearly identical PFS (HR 0.955, 95%CI 0.515–1.77, *p* = 0.884), although the IT regimen was associated with a lower occurrence of SAEs in contrast to the IC regimen (*p* < 0.05). In addition, the IT regimen demonstrated superior PFS (HR 0.583, 95%CI 0.345–0.985, *p* = 0.044) and fewer SAEs when compared to C alone (*p* < 0.05). These findings collectively support the notion that the combination of ICIs, target and chemotherapy exhibits robust antitumor activity in previously treated R/M NPC patients, without a significant increase in adverse events.

## Introduction

Nasopharyngeal carcinoma (NPC) stands apart due to its unique etiological and geographical distribution when compared to other head and neck squamous cell carcinomas (HNSCC). It exhibits a pronounced geographic concentration, with a high prevalence in South China and Southeast Asia. Among its various pathological subtypes, nonkeratinizing NPC, closely linked to Epstein-Barr virus (EBV) infection, is the most prevalent one^1–3^. Approximately 6–15% of newly diagnosed NPC patients present with distant metastasis, and relapse or metastasis occurs in about 20–40% of NPC patients initially diagnosed without metastasis, who subsequently receive systemic therapy^4^. For patients with recurrent or metastatic NPC (R/M NPC), platinum-based chemotherapy is the established first-line treatment, yet their median overall survival (OS) remains under 20 months^5^. Unfortunately, there is no universally recognized treatment option for those who failed to first-line chemotherapy, and their prognosis is dismal, with a median OS of approximately 12 months^5,6^.

Recent advancements in imaging and radiotherapy technology, along with the emergence of immunotherapy, have improved the survival prospects of R/M NPC patients. Notably, EBV-induced NPC, characterized by overexpression of programmed cell death-ligand 1 (PD-L1) and significant lymphocyte infiltration, shows promise as a candidate for immunotherapy^7^. Multiple studies involving R/M NPC patients treated with anti-PD-1 immune checkpoint inhibitors (ICIs) have demonstrated robust activity with an objective response rate (ORR) ranging from 20 to 34%^8–12^. However, only a subset of R/M NPC patients benefit from ICIs after failing first-line therapy and their prognosis remains bleak. Therefore, it is of significant importance to identify more effective treatments for this patient group.

A growing body of research has indicated that anti-angiogenic agents can not only normalize tumor blood vessels, but also obstruct immunosuppressive signals in tumors. In parallel, ICIs can reinstate an immune supportive microenvironment and promote vascular normalization. These findings provide a compelling rationale for the application of anti-angiogenesis in conjunction with ICIs^13,14^. Besides, anti-epidermal growth factor receptor (EGFR) therapy can enhance immune cell interactions by triggering antibody-dependent cellular cytotoxicity, leading to an augmented antitumor effect^15–17^. Given that ICIs can facilitate immune rejection and tumor regression by augmenting cytotoxic lymphocytes, the combination of anti-EGFR therapy with ICIs may yield a synergistic antitumor effect. An array of studies has illuminated the promising potential of ICIs when combined with other target agents in conditions such as non-small cell lung cancer, ovarian cancer, and HNSCC^18–21^. Nonetheless, currently there is no study focusing on the combination of ICIs with target therapy (anti-angiogenesis or EGFR inhibitors) in the treatment of R/M NPC.

Therefore, we have designed this retrospective study involving previously treated R/M NPC patients to investigate the potential treatment efficacy of combining ICIs and target therapy, with or without traditional chemotherapy. We aimed to compare these combined regimens to chemotherapy, with or without ICIs, in a real-world clinical context.

## Results

### Patient characteristics

A total of 226 R/M NPC patients who had previously experienced treatment failure with at least first line salvage therapy, were enrolled in this study, distributed across 4 distinct treatment regimens. The median follow-up period encompassed13.1 months (range 1.4–47.4) for all participants. Disease progression occurred in 136 patients, and 47 patients died up to the latest follow-up. The cohort exhibited a median age of 46 years, spanning from 18 to 70 years, with a male-to-female ratio of 3.4: 1. Notably, no statistically significant differences were observed in patient characteristics among the various treatment groups. A comprehensive summary of patient characteristics is available in Table 1 and Table S1.

**Table 1:** Characteristics of the 226 patients.

	IC + C (n = 108)	ITC + IT (n = 118)	*p* value
Age			0.535
< 50	72	74	
≥ 50	36	44	
Sex			0.841
Male	83	92	
Female	25	26	
NPC family history			0.604
Yes	8	11	
No	100	107	
KPS score			0.918
70–80	25	28	
90–100	83	90	
BMI			0.818
< 18.5	21	20	
18.5–25	73	80	
> 25	14	18	
Disease status			0.519
Recurrent	27	34	
Metastatic	81	84	
Treatment line			0.268
2	70	68	
≥ 3	38	50	
Number of metastatic sites			0.774
0	27	34	
1–2	13	15	
≥ 3	68	69	
EBV DNA level			0.213
Positive	83	82	
Negative	25	36	
Metastatic site			
Liver	41	36	0.238
Lung	32	31	0.574
Bone	45	51	0.813
Other	19	25	0.496

*ITC* ICIs with anti-angiogenesis or EGFR inhibitor therapy and chemotherapy, *IT* ICIs with anti-angiogenesis or EGFR inhibitor therapy, *IC* ICIs with chemotherapy, *C* chemotherapy, *NPC* nasopharyngeal carcinoma, *KPS* Karnofsky performance status, *BMI* body mass index, *EBV* Epstein-Barr virus.

**Figure 1:**
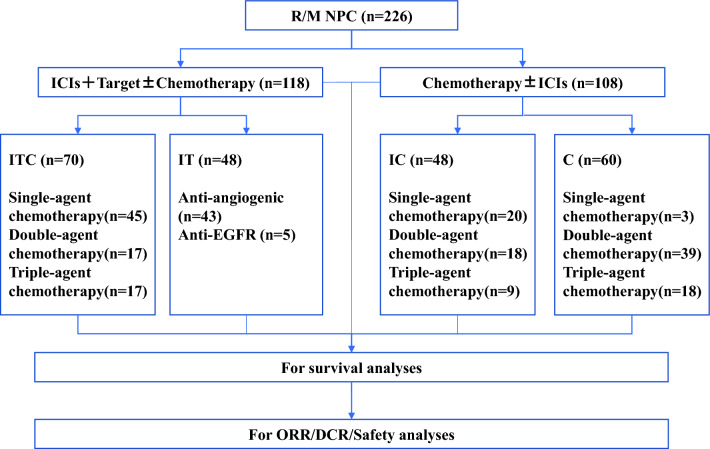
Treatment regimen of R/M NPC patients who failed to at least first line salvage therapy. R/M NPC: recurrent or metastatic nasopharyngeal carcinoma; ICIs: immune checkpoint inhibitors; EGFR: anti-epidermal growth factor receptor; ORR: objective response rate; DCR: disease control rate; ITC: ICIs with anti-angiogenesis or EGFR inhibitor therapy and chemotherapy; IT: ICIs with anti-angiogenesis or EGFR inhibitor therapy; IC: ICIs with chemotherapy; C: chemotherapy.

Among the participants, 70 patients received the ITC regimen, 48 patients received the IT regimen, 48 patients were treated with IC, and the remaining 60 patients treated with chemotherapy alone (Fig. 1). In the ITC, IC, and C treatment groups, 45, 20, and 3 patients received single-agent chemotherapy, respectively. Moreover, 17, 18, and 39 patents in these respective groups received double-agent chemotherapy, while 17, 9, and 18 patients underwent triple-agent chemotherapy, respectively.

### ICIs in combination with target therapy (anti-angiogenesis or EGFR inhibitors) with or without chemotherapy (ITC/IT) versus chemotherapy with or without ICIs (IC/C)

**Figure 2:**
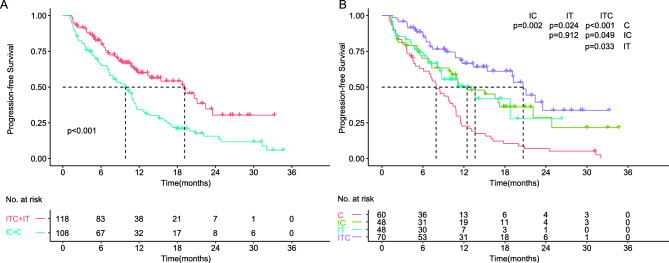
Kaplan–Meier PFS curves of R/M NPC patients who failed at least first line salvage therapy.

**Figure 3:**
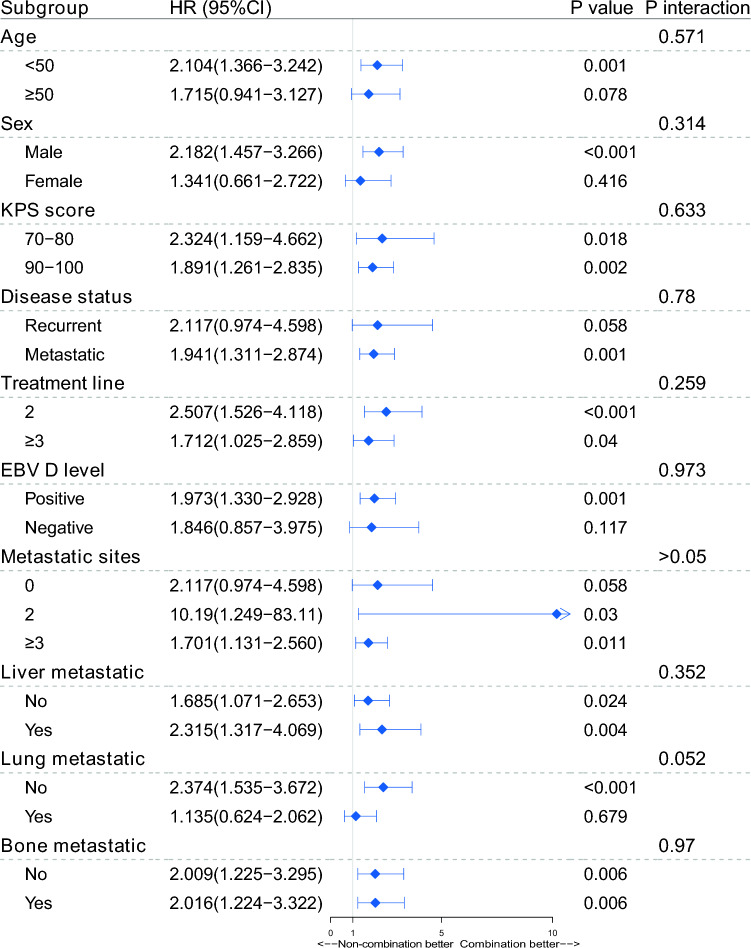
Forest plot for key subgroup analyses of PFS, comparing IC/C treatment regimen to ITC/IT treatment regimen. HR: hazard ratio; CI: confidence interval; KPS: Karnofsky performance status; EBV: Epstein-Barr virus.

The median progression-free survival (PFS) was 19.1 months for the ITC/IT group and 9.8 months for the IC/C group (Fig. 2a). Patients receiving IC/C treatment exhibited a notably poorer PFS (HR 2.01, 95% CI 1.411–2.865, *p* < 0.001, Fig. 2a, Table S2), when compared to the ITC/IT group. An enhancement in PFS was observed in the ITC/IT treatment group across almost all subgroups, with no discernible significant interaction effect between treatment subgroups (all *p* > 0.05, Fig. 3). As shown in Fig. 3, patients with positive EBV DNA level significantly benefit from ITC/IT treatment compared to IC/C treatment, while the others did not. Likewise, the advantages of the ITC/IT regimen are more pronounced in R/M NPC patients who are younger, male, or with metastatic, compared to the corresponding patients (all *p* < 0.05, Fig. 3). The median OS results for the ITC/IT group could not be reliably determined, as they were still evolving, with a median OS of 32.567 months observed for the IC/C group.

Table 2 displays the distribution of patients achieving complete response (CR), partial response (PR), stable disease (SD), and progressive disease (PD). In aggregate, the ORR in the ITC/IT group was significantly higher (43.22%) than that in the IC/C group (29.63%, *p* = 0.034). The disease control rate (DCR) was 88.98% in the ITC/IT group and 82.41% in the IC/C group (*p* = 0.157).

**Table 2:** Summary of tumor response for the current study.

Best overall response, n (%)	IC + C	ITC + IT	*p* value
ORR	32 (29.63%)	51 (43.22%)	**0.034**
CR	5	7	
PR	27	44	
SD	57	54	
PD	19	13	
DCR	89 (82.41%)	105 (88.98%)	0.157

*ORR* objective response rate, *CR* complete response, *PR* partial response, *SD* stable disease, *PD* progressive disease, *DCR* disease control rate. Significant values are in bold.

Importantly, there were no occurrences of treatment-related mortality during the course of the treatments administered. Anemia and leukopenia emerged as the most common treatment-related adverse events (AEs) for both groups, as indicated in Table 3). Although there were no significant differences in the incidence of AEs of any grade (*p* = 0.284), patients in the IC/C group experienced a higher frequency of severe adverse events (SAEs) (*p* = 0.009).

**Table 3:** Treatment-related adverse events of all patients.

	Any grade		*p* value	Grade ≥ 3		*p* value
	IC + C	ITC + IT		IC + C	ITC + IT	
Leukopenia	64	57		24	6	
Neutropenia	59	33		14	7	
Anemia	87	72		13	6	
Thrombocytopenia	38	26		9	6	
CRE	24	21		0	0	
TBIL	2	18		0	2	
ALT	24	22		1	1	
AST	23	24		2	4	
ALP	7	20		2	0	
Rash	12	5		0	2	
Nausea	49	38		0	0	
Vomit	18	13		0	0	
Hand-foot syndrome	13	15		1	0	
Capillary hyperplasia	2	8		0	1	
Myocarditis	2	1		0	1	
Pneumonia	0	2		0	1	
Hypothyroidism	11	27		0	0	
Musculoskeletal pain	5	8		0	1	
Nasopharyngeal necrosis	4	7		0	0	
Epistaxia	0	9		0	1	
Mucositis oral	1	9		0	1	
Headache	2	8		0	1	
Total	102 (94.44%)	107 (90.7%)	0.284	40 (37.03%)	25 (20.34%)	**0.009**

*CRE* creatinine, *TBIL* total bilirubin, *ALT* alanine transaminase, *AST* aspartate aminotransferase, *ALP* alkaline phosphatase. Significant values are in bold.

### ICIs in combination with target therapy (anti-angiogenesis or EGFR inhibitors) and chemotherapy (ITC) versus ICIs in combination with target therapy (IT) and chemotherapy with or without ICIs (IC/C)

The median PFS periods were as follows: 20.7 months in the ITC cohort, 13.6 months in the IT cohort, 12.5 months in the IC cohort, and 7.9 months in the C cohort (Fig. 2b). Notably, the ITC cohort demonstrated superior PFS compared to the other three cohorts (ITC vs. IT, HR 0.465, 95%CI 0.252–0.855, *p* = 0.014; ITC vs. IC, HR 0.561, 95%CI 0.323–0.972, *p* = 0.039; ITC vs. C, HR 0.288, 95%CI 0.178–0.464, *p* < 0.01). There was no substantial interaction effect observed between the cohorts and subgroup characteristics (Figure S1). For median OS, a value of 30.6 months was noted for the C cohort, while the results for the ITC, IT, and IC cohorts were not yet mature. Importantly, the ITC cohort exhibited a significantly improved OS compared to the C cohort (HR 0.336, 95%CI 0.123–0.915, *p* = 0.033, Table S2).

There was no statistically significant difference in ORR, DCR, and treatment-related AEs between the ITC and IT or IC cohorts (all *p* > 0.05, Table S3–4). However, when compared to the C cohort, the ITC cohort displayed a significantly enhanced ORR (48.57% vs. 25%, *p* = 0.006) and DCR (82.86% vs. 80%, *p* = 0.03).

### ICIs in combination with target therapy (anti-angiogenesis or EGFR inhibitors) (IT) versus chemotherapy with or without ICIs (IC/C)

The IT cohort did not exhibit a significantly improved PFS compared to the IC cohort (HR 0.955 95%CI 0.515–1.77, *p* = 0.884), but it did fare better than the C cohort (HR 0.583 95%CI 0.345–0.985, *p* = 0.044). Importantly, there was no observable significant interaction effect between the cohorts and the characteristics of the subgroups (Figure S1). Moreover, there were no statistically significant differences in terms of ORR, DCR, or the incidence of treatment-related AEs of any grade between the IT and IC cohorts and the C cohorts (Tables S3–4). However, the IT cohort was associated with a significantly lower occurrence of SAEs in comparison to both the IC and C cohorts (*p* = 0.018 and *p* = 0.006 respectively).

## Discussion

For patients with R/M NPC who failed first-line chemotherapy, treatment and options are limited. Depending on the disease stage and patient condition, salvage strategies encompass surgery, chemotherapy, radiotherapy, ICIs, chemotherapy combined with EGFR inhibitors, and chemotherapy combined with ICIs. Though the usage of immune or target therapy improved patients’ outcomes recent years, these patients generally have a poor prognosis. Single-agent chemotherapy, considered a standard subsequent-line treatment, exhibited a median PFS of 5.5 months and ORR of 23.3%^21^, while immune checkpoint inhibitors exhibited similar antitumor efficacy, with a median PFS of 1.9–6.5 months and ORR of 20.5–34%^8–12^. Given the persistently bleak prognosis, the quest for novel treatment regimens to potentially prolong survival remains an urgent need.

An open-label, multicenter, phase 2 trial evaluated the antitumor activity of a combination of ICIs and EGFR inhibitor for R/M HNSCC and the results showed promising clinical activity with ORR of 45%^22^. Recently, several studies have reported the antitumor efficacy of ICIs in combination with anti-angiogenesis in R/M NPC patients who failed at least first line therapy, with a median PFS of 4.5–10.4 months and an ORR of 33.3–65.5%^23–26^. Our recent study demonstrated that R/M NPC patients who had progressed from prior anti-PD1 therapy could benefit from the anti-PD1 rechallenge in combination with anti-angiogenesis or anti-EGFR agents with or without chemotherapy^27^. These findings collectively underscore the rationale and promising potential of combining immunotherapy with target therapy for R/M NPC patients who failed to at least first line therapy, even those who failed to prior immune therapy. However, this approach warrants validation through further phase 3 trials.

In our present study, the combination of ICIs with anti-angiogenesis or EGFR inhibitor and chemotherapy (ITC) treatment achieved the most favorable PFS among the four treatment regimens (Fig. 2b, Table S2). The ICIs in combination with anti-angiogenesis or EGFR inhibitor (IT) cohort achieved a PFS similar to that seen in the cohort of chemotherapy with ICIs (IC), but significantly longer than chemotherapy (C) cohort. Chemotherapy alone showed the worst outcome for these patient population taking survival, tumor response and safety all into consideration (Fig. 2b, Table S2–4). It is worth noting that only EBV DNA level positive patients exhibited significant better PFS in ITC/IT group compared to IC/C group (Fig. 3), which was similar to the result from CAPTAIN-1st study that R/M patients with positive EBV DNA level could benefit from immune-combined therapy significantly while the others did not^28^. This hints us that the combination of immunotherapy with target therapy with or without chemotherapy may be superior to the chemotherapy with or without immunotherapy only in EBV DNA level positive population. Anti-angiogenesis and anti-EGFR inhibitors possess disparate mechanisms of anti-tumor action. Given that target therapy enrolled in this study was mainly anti-angiogenesis inhibitors (ITC cohort, n = 52; IT cohort, n = 43), we further excluded the anti-EGFR patients (ITC cohort, n = 18; IT cohort, n = 5), and K–M curves of PFS are shown in Figure S2. The ITC/IT cohort still had a significant PFS compared to the IC/C cohort, and the ITC cohort still had the longest median PFS among the four specific groups. The ITC and IT cohorts achieved ORRs of 48.57% and 35.42%, and DCR of 82.86% and 83.33%, respectively (Table S3). The promising anti-tumor activity of the combination treatment may be the result of the synergistic effect between ICIs and targeted drugs, although the precise underlying mechanism necessitates further exploration.

With regard to treatment-related AEs, no significant differences in the incidence of AEs of any grade were observed among the four cohorts. Notably, the IT cohort appeared to be safer, with significantly fewer SAEs compared to the IC and C cohorts, a likely consequence of the absence of chemotherapy. It is worth noting that any grade AEs or SAEs in ITC cohort did not increase significantly than other cohorts, which can be attributed to the cut down usage of chemotherapy. As shown in Fig. 1, according to the combination of ICIs and target therapy, more than half of patients received single-agent chemotherapy while almost all patients in C group got two or more agent chemotherapy. Additionally, the heightened attention to patients as treatment was intensified in the ITC cohort may contribute to this phenomenon. Although it looks like that ITC regimen bring more immune-related SAEs, such as myocarditis and pneumonia (Table S4), it was manageable, of which was similar to the results of a recent study^29^. Therefore, it may be wise to combine immunotherapy and targeted therapy by reducing the number of chemotherapy drugs to achieve the purpose of improving efficacy and decreasing toxicity.

Several limitations need to be acknowledged in this study. Firstly, it was a retrospective study with all enrolled patients coming from a single-center and endemic region characterized by a predominance of undifferentiated non-keratinizing carcinoma. The efficacy of ITC or IT treatment regimens in other regions, where keratinizing carcinoma is more prevalent, remains to be confirmed. Secondly, the primary endpoint was not OS due to the immaturity of the data and additional time is required to assess its impact on OS. Furthermore, the median follow-up period was relatively short, and a longer duration is necessary to corroborate the findings presented in this paper. Thirdly, this was a real-world study, and there was inevitably some heterogeneity as neither the ICIs agents nor the targeted agents or chemotherapy agents were uniform. Nevertheless, the objective of this study was to explore a more promising combination regimen compared to conventional chemotherapy for previously treated recurrent or metastatic nasopharyngeal carcinoma in the real world, as aspect which had not been investigated previously. We believe that the results of this study have certain guiding significance for the future clinical research direction. Finally, the description of AEs, especially for immune-related AEs, was not comprehensive, reflecting the limitation inherent to retrospective studies. Currently, we are conducting a prospective study investigating the clinical effects of the combination of triplimab and nimotuzumab for the treatment of high risk locally advanced NPC (ChiECRCT20220133), and another prospective study evaluating the feasibility of combining camrelizumab and apatinib in the treatment of high risk locally advanced NPC (ChiCTR2000032317). The results of these studies may further substantiate the efficacy and safety of the combination of ICIs and target therapy in NPC patients.

## Conclusion

The combination therapy involving ICIs and targeted therapy (anti- angiogenesis or EGFR inhibitors) as a second line or subsequent treatment for R/M NPC has exhibited the ability to extend PFS without a significant increase in AEs. Notably, among the regimens studied, ITC demonstrated the most favorable results, with IT surpassing IC due to its lower incidence of SAEs, and outperforming C in terms of both PFS and SAEs. In summary, our findings underscore the potential of the combination of ICIs and target therapy as a novel and improved therapeutic option for previously treated R/M NPC, leading to a notable extension of their PFS. Subsequent evaluation of this combination therapy in prospective, multicenter, meticulously designed studies with larger sample sizes is imperative.

## Materials and methods

### Ethics statement

This study was approved by the Ethics Committee of Sun Yat-Sen University Cancer Center (SYSUCC), with Institution Review Board (IRB) Number of B2023-245-01 on May 25th, 2023. Informed consent was dispensed with, owing to the retrospective nature of this study and the anonymization of patient data, a decision that received the approval of the IRB at Sun Yat-Sen University Cancer Center (SYSUCC). The entirety of the study’s methods and procedures was executed in strict adherence to the guidelines established by the Institutional Review Boards of SYSUCC.

### Patient selection

Previously treated R/M NPC patients at Sun Yat-sen University Cancer Center (SYSUCC) between January 1, 2019 and December 30, 2021 were included in this real-world clinical practice study. Eligibility criteria included: pathologically confirmed or radiologically diagnosed R/M NPC; failed at least first line treatment; age between 18 and 80 years; Karnofsky performance status (KPS) ≥ 70; adequate organ function; undergone treatment regimens involving ICIs in combination with target therapy (anti-angiogenesis or EGFR inhibitors) and chemotherapy (ITC), or ICIs in combination with target therapy (IT), or ICIs in combination with chemotherapy (IC), or chemotherapy (C) alone as following treatment; and with regular imaging evaluations. Exclusion criteria included: known other malignancies; active autoimmune disease; received surgery; did not received systemic therapy after progression; had incomplete information.

### Treatment

Based on the afore mentioned treatments, the enrolled patients were divided into four distinct groups, denoted as the ITC group, IT group, IC group, and C group. Among these groups, the most commonly chemotherapy regimens involved gemcitabine/cisplatin (GP), docetaxel/cisplatin/capecitabine (TPC), docetaxel/cisplatin/5-fluorouracil (TPF), and cisplatin/5-fluorouracil (PF). ICIs included terriprizumab, camrelizumab, pembrolizumab, nivolumab, cindillizumab, and tislelizumab. Anti-angiogenesis agents included bevacizumab, anlotinib, apatinib, and endostar. Additionally, EGFR inhibitors included cetuximab and nimotuzumab. All regimens were administered every 3 weeks until disease progression, intolerable toxicity, consents withdrawal or physician’s decision. All enrolled patients received at least two cycles of their respective treatment.

### Outcome assessment

Radiological evaluations were conducted every two to three treatment cycles, and laboratory tests were administered prior to the initiation of each treatment cycle. Tumor response was appraised utilizing RECIST V1.1 and the immune-related RECIST criteria. Adverse events were recorded using National Cancer Institute Common Terminology Criteria (CTCAE) version 4.0. The primary endpoint was PFS, defined as time from administration of treatment to disease progression or death, whichever occurred first. The second endpoints encompassed OS, ORR, DCR, and safety assessments.

### Statistical analysis

Differences in clinical characteristics, tumor response, and AEs among the groups were examined through the application of the Chi-square test. The analysis of PFS and OS was executed using Kaplan–Meier method, with the assessment of survival distinctions being conducted through the log-rank test. Interaction analyses between the groups and clinical variables were performed, and a stratified Cox proportional hazards model was employed to compare the groups for PFS. Multivariable analyses of characteristics were performed using the Cox proportional hazards model. Statistical Package for Social Sciences (version 21.0; IBM Corp.) and R (http://www.R-project.org) software were used for all analyses, and *p* < 0.05 was considered statistically significant.

### Supplementary Information

Supplementary Information.

## Data Availability

All original data are presented in the article and supplementary material. Further inquiries can be obtained from the corresponding author.
