# Supplementary material for: Combination strategy exploration for prior treated recurrent or metastatic nasopharyngeal carcinoma in the era of immunotherapy
Source: Sci Rep. 2024 Jan 20;14:1768. doi: 10.1038/s41598-024-52326-7 (PMC10798952; doi:10.1038/s41598-024-52326-7)
Supplement: Supplementary file 1 — Supplementary Information. [file 41598_2024_52326_MOESM1_ESM.docx]

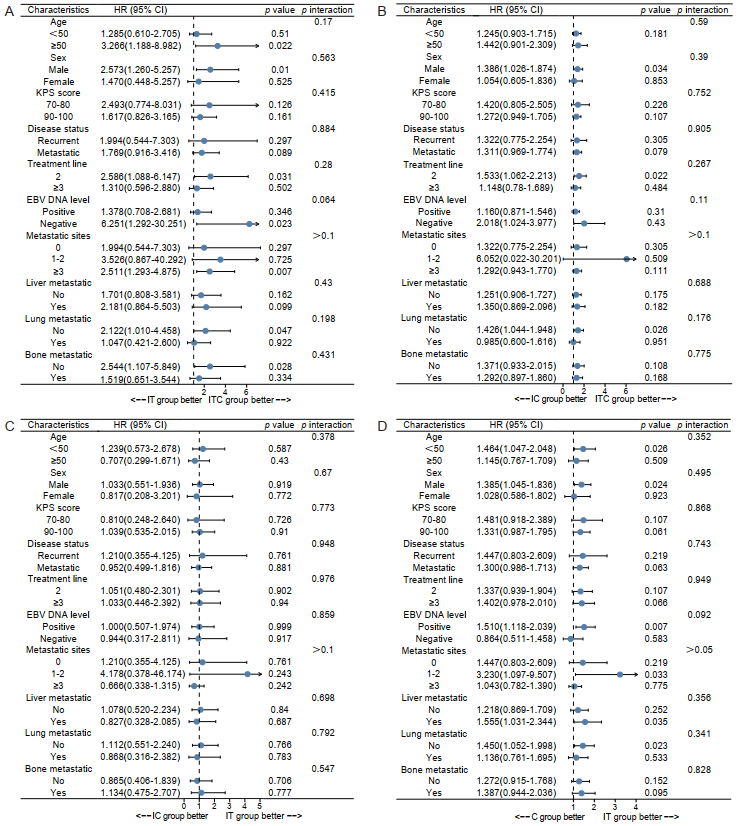


Figure S1: Forest plot for key subgroup analyses of PFS for the four cohorts **A):** IT vs ITC; **B)** IC vs ITC; **C)** IC vs IT; **D)** C vs IT. ITC: ICIs in combination with target therapy (anti-angiogenesis or EGFR inhibitors) and chemotherapy; IT: ICIs in combination with target therapy; IC: ICIs in combination with chemotherapy; C: chemotherapy alone. HR: hazard ratio; CI: confidence interval; KPS: Karnofsky performance status; EBV: Epstein-Barr virus.


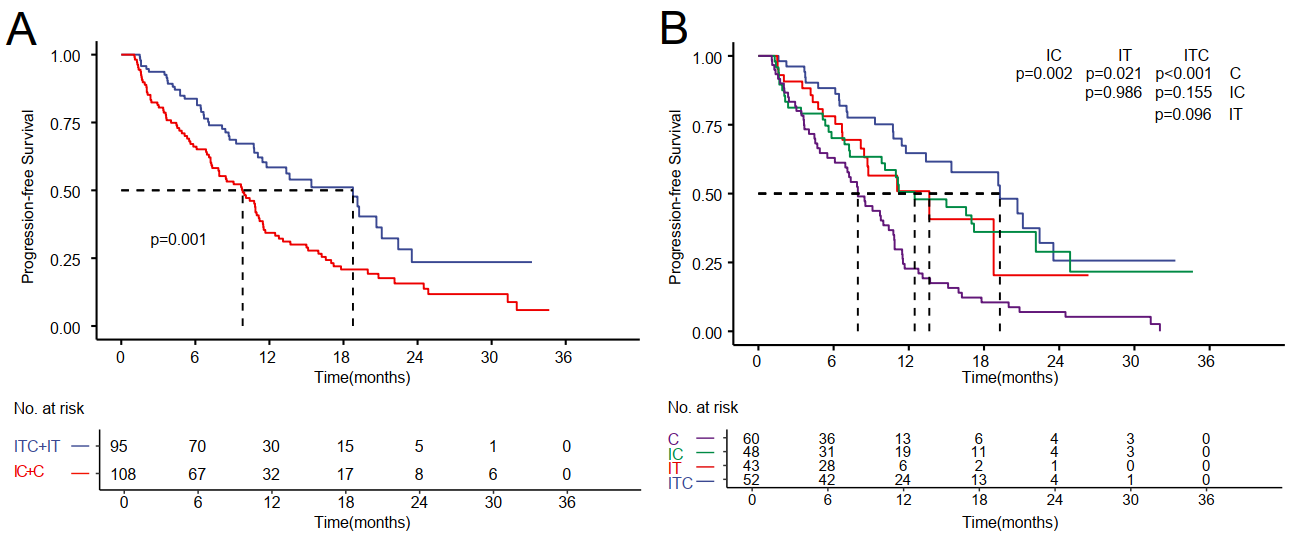


Figure S2: Kaplan-Meier PFS curves of R/M NPC patients who failed at least first line salvage therapy after exclusion of anti-EGFR patients.

Table S1. Detailed characteristics of all patients.

|  | IC (n=48) | C (n=60) | ITC (n=70) | IT (n=48) | P value |
| --- | --- | --- | --- | --- | --- |
| Age |  |  |  |  | 0.362 |
| ＜50 | 28 | 44 | 45 | 29 |  |
| ≥50 | 20 | 16 | 25 | 19 |  |
| Sex |  |  |  |  | 0.327 |
| Male | 40 | 43 | 52 | 40 |  |
| Female | 8 | 17 | 18 | 8 |  |
| KPS score |  |  |  |  | 0.551 |
| 70-80 | 8 | 17 | 16 | 12 |  |
| 90-100 | 40 | 43 | 54 | 36 |  |
| BMI |  |  |  |  | 0.968 |
| ＜18.5 | 10 | 11 | 11 | 9 |  |
| 18.5-25 | 31 | 42 | 47 | 33 |  |
| ＞25 | 7 | 7 | 12 | 6 |  |
| Disease status |  |  |  |  | 0.545 |
| Recurrent | 15 | 12 | 20 | 14 |  |
| Metastatic | 33 | 48 | 50 | 34 |  |
| Treatment line |  |  |  |  | 0.494 |
| 2 | 32 | 38 | 43 | 25 |  |
| ≥3 | 16 | 22 | 27 | 23 |  |
| Metastatic sites |  |  |  |  | 0.098 |
| 0 | 15 | 12 | 20 | 14 |  |
| 1-2 | 6 | 7 | 4 | 11 |  |
| ≥3 | 27 | 41 | 46 | 23 |  |
| Metastatic site |  |  |  |  |  |
| Liver | 15 | 26 | 23 | 13 | 0.314 |
| Lung | 10 | 22 | 17 | 14 | 0.268 |
| Bone | 19 | 26 | 33 | 18 | 0.731 |
| Other | 8 | 11 | 11 | 14 | 0.284 |
| EBV DNA level |  |  |  |  | 0.295 |
| Positive | 36 | 47 | 52 | 30 |  |
| Negative | 12 | 13 | 18 | 18 |  |
| Heart disease |  |  |  |  | 0.293 |
| No | 47 | 60 | 70 | 48 |  |
| Yes | 1 | 0 | 0 | 0 |  |
| Hypertension |  |  |  |  | 0.093 |
| No | 47 | 56 | 60 | 45 |  |
| Yes | 1 | 4 | 10 | 3 |  |
| Diabetes |  |  |  |  | 0.178 |
| No | 47 | 60 | 70 | 46 |  |
| Yes | 1 | 0 | 0 | 2 |  |
| Hepatitis B |  |  |  |  | 0.63 |
| No | 43 | 54 | 65 | 41 |  |
| Yes | 5 | 6 | 5 | 7 |  |
| Smoking |  |  |  |  | 0.237 |
| No | 33 | 51 | 53 | 38 |  |
| Yes | 15 | 9 | 17 | 10 |  |
| Drink |  |  |  |  | 0.293 |
| No | 40 | 55 | 63 | 39 |  |
| Yes | 8 | 5 | 7 | 9 |  |
| Tumor family history |  |  |  |  | 0.872 |
| No | 40 | 47 | 54 | 38 |  |
| Yes | 8 | 13 | 16 | 10 |  |
| NPC family history |  |  |  |  | 0.487 |
| No | 46 | 54 | 62 | 45 |  |
| Yes | 2 | 6 | 8 | 3 |  |

ITC: ICIs with anti-angiogenesis or EGFR inhibitor therapy and chemotherapy; IT: ICIs with anti-angiogenesis or EGFR inhibitor therapy; IC: ICIs with chemotherapy; C: chemotherapy; KPS: Karnofsky performance status; BMI: body mass index; EBV: Epstein-Barr virus; NPC: nasopharyngeal carcinoma.

Table S2: Multi-cox analysis for PFS and OS in detailed cohorts.

|  | PFS | |  | OS | |
| --- | --- | --- | --- | --- | --- |
| Variable | HR (95% CI) | P value |  | HR (95% CI) | P value |
| Regime (IC/C vs ITC/IT） | 2.01(1.411-2.865) | **＜0.001** |  | 1.829(0.883-3.785) | 0.104 |
| Regime (ITC vs IT) | 0.465(0.252-0.855) | **0.014** |  | 0.769(0.175-3.372) | 0.728 |
| Regime (ITC vs IC) | 0.561(0.323-0.972) | **0.039** |  | 0.486(0.144-1.635) | 0.244 |
| Regime (IT vs IC) | 0.955(0.515-1.77) | 0.884 |  | 1.393(0.323-6.008) | 0.657 |
| Regime (IT vs C) | 0.583(0.345-0.985) | **0.044** |  | 0.769(0.267-2.217) | 0.626 |
| Regime (ITC vs C) | 0.288(0.178-0.464) | **＜0.001** |  | 0.336(0.123-0.915) | **0.033** |
| Regime (IC vs C) | 0.481(0.297-0.779) | **0.003** |  | 0.521(0.226-1.2) | 0.125 |

HR: hazard ratio; CI: confidence interval. All p value were adjusted by age, sex, KPS score, disease status, prior lines, EBV DNA level, liver metastatic, bone metastatic, lung metastatic, and other metastatic.

Table S3. Summary of tumor response for the current study in detailed cohorts.

|  | ITC | IT | IC | C |  | ORR | ITC | IT | IC |  |  |  |
| --- | --- | --- | --- | --- | --- | --- | --- | --- | --- | --- | --- | --- |
| ORR | 34(48.57%) | 17(35.42%) | 17(35.42%) | 15(25%) |  | C | **0.006** | 0.239 | 0.239 |  |  |  |
| CR | 6 | 1 | 3 | 2 |  | IC | 0.156 | 1.0 |  |  |  |  |
| PR | 28 | 16 | 14 | 13 |  | IT | 0.156 |  |  |  | 0.105 | IT |
| SD | 31 | 23 | 24 | 33 |  |  |  |  |  | 0.779 | 0.189 | IC |
| PD | 5 | 8 | 7 | 12 |  |  |  |  | 0.463 | 0.658 | **0.030** | C |
| DCR | 65(82.86%) | 40(83.33%) | 41(85.42%) | 48(80%) |  |  |  |  | IC | IT | ITC | DCR |

ORR: objective response rate; CR: complete response; PR: partial response; SD: stable disease; PD: progressive disease; DCR: disease control rate.

Table S4: Treatment-related adverse events of all patients in detailed cohorts.

|  | Any Grade | | | |  | Grade≥3 | | | |  |  |  |  |
| --- | --- | --- | --- | --- | --- | --- | --- | --- | --- | --- | --- | --- | --- |
|  | ITC | IT | IC | C |  | ITC | IT | IC | C | AnyG | ITC | IT | IC |
| Leukopenia | 37 | 20 | 26 | 36 |  | 5 | 1 | 8 | 15 | C | 0.612 | 0.161 | 0.778 |
| Neutropenia | 24 | 9 | 23 | 34 |  | 6 | 1 | 5 | 9 | IC | 0.85 | 0.294 |  |
| Anemia | 49 | 23 | 38 | 46 |  | 5 | 1 | 6 | 7 | IT | 0.325 |  |  |
| Thrombocytopenia | 23 | 3 | 12 | 25 |  | 5 | 1 | 5 | 4 |  |  | 0.146 | IT |
| CRE | 9 | 12 | 7 | 16 |  | 0 | 0 | 0 | 0 |  | **0.018** | 0.318 | IC |
| TBIL | 16 | 2 | 1 | 1 |  | 2 | 0 | 0 | 0 | 0.755 | **0.006** | 0.159 | C |
| ALT | 13 | 9 | 12 | 12 |  | 0 | 1 | 1 | 0 | IC | IT | ITC | SAEs |
| AST | 13 | 11 | 11 | 12 |  | 2 | 2 | 1 | 1 |  |  |  |  |
| ALP | 10 | 10 | 2 | 3 |  | 0 | 0 | 1 | 1 |  |  |  |  |
| Rash | 9 | 3 | 3 | 2 |  | 1 | 1 | 0 | 0 |  |  |  |  |
| Nausea | 28 | 10 | 21 | 28 |  | 0 | 0 | 0 | 0 |  |  |  |  |
| Vomit | 9 | 4 | 7 | 11 |  | 0 | 0 | 0 | 0 |  |  |  |  |
| Hand-foot syndrome | 10 | 5 | 4 | 9 |  | 0 | 0 | 0 | 1 |  |  |  |  |
| Capillary hyperplasia | 4 | 4 | 2 | 0 |  | 1 | 0 | 0 | 0 |  |  |  |  |
| Myocarditis | 1 | 0 | 2 | 0 |  | 1 | 0 | 0 | 0 |  |  |  |  |
| Pneumonia | 1 | 1 | 0 | 0 |  | 1 | 0 | 0 | 0 |  |  |  |  |
| Hypothyroidism | 15 | 12 | 11 | 0 |  | 0 | 0 | 0 | 0 |  |  |  |  |
| Musculoskeletal pain | 5 | 3 | 3 | 2 |  | 1 | 0 | 0 | 0 |  |  |  |  |
| Nasopharyngeal necrosis | 2 | 5 | 3 | 1 |  | 0 | 0 | 0 | 0 |  |  |  |  |
| Epistaxia | 6 | 3 | 0 | 0 |  | 1 | 0 | 0 | 0 |  |  |  |  |
| Mucositis oral | 3 | 6 | 0 | 1 |  | 0 | 1 | 0 | 0 |  |  |  |  |
| Headache | 6 | 2 | 0 | 2 |  | 1 | 0 | 0 | 0 |  |  |  |  |

CRE: creatinine; TBIL: total bilirubin; ALT: alanine transaminase; AST: aspartate aminotransferase; ALP: alkaline phosphatase.
